# Rückkehr an den Arbeitsplatz von Beschäftigten nach einer psychischen Erkrankung: Entwicklung eines Leitfadens und eines Schulungsmoduls für Betriebsärztinnen/Betriebsärzte

**DOI:** 10.1007/s40664-022-00471-z

**Published:** 2022-06-12

**Authors:** Jessica Scharf, Adrian Loerbroks, Peter Angerer

**Affiliations:** Institut für Arbeits‑, Sozial- und Umweltmedizin, Centre for Health and Society, Medizinische Fakultät, Heinrich-Heine-Universität Düsseldorf, Moorenstr. 5, 40225 Düsseldorf, Deutschland

**Keywords:** Psychische Erkrankung, Betriebliches Eingliederungsmanagement, Betriebsarzt, Partizipative Entwicklung, Leitfaden, Mental disorder, Return to work management, Occupational physician, Participatory development, Guidelines

## Abstract

**Zielstellungen:**

Die Rückkehr an den Arbeitsplatz scheitert häufig an widersprüchlichen Erwartungen und Zielen der beteiligten Akteure. Aufgrund ihrer medizinischen Expertise und arbeitsplatzbezogenen Kenntnisse könnten Betriebsärzte bzw. Betriebsärztinnen gegenseitiges Verständnis und Zusammenarbeit der Beteiligten effektiv fördern. Es soll ein Leitfaden und eine Schulung zu dessen Anwendung entwickelt werden, die Betriebsärzte bzw. Betriebsärztinnen unterstützen, eine vermittelnde Rolle bei der Eingliederung psychisch erkrankter Beschäftigter einzunehmen.

**Methoden:**

Basierend auf einer Literaturrecherche sowie umfangreichen qualitativen Vorarbeiten zu den Erwartungen der Akteure, wurden in mehreren konsekutiven Schritten ein Leitfaden und Schulungsmaterialien partizipativ mit Arbeitsmedizinerinnen und Arbeitsmedizinern und anderen Experten bzw. Expertinnen entwickelt, diskutiert und überarbeitet. Schließlich wurden Betriebsärzte und Betriebsärztinnen in Weiterbildungskursen in der Anwendung des Leitfadens geschult, um ihn anschließend im Arbeitsalltag auf Praktikabilität zu überprüfen.

**Ergebnisse:**

Der Leitfaden informiert über die potenziell unterschiedlichen Erwartungen der Akteure bzw. Akteurinnen an den Rückkehrprozess und bietet umfassende Handlungshilfen für die vermittelnde Arbeit des Betriebsarztes/der Betriebsärztin. Nach der etwa zweistündigen manualisierten Schulung setzten innerhalb von 4 Monaten 9 von 37 Teilnehmern den Leitfaden für Rückkehrgespräche ein, 6 von 9 bewerteten ihn als hilfreich.

**Schlussfolgerung:**

Die ersten positiven Anwendungserfahrungen des mit der Zielgruppe entwickelten Materials rechtfertigt eine größere Interventionsstudie, um den zu vermutenden positiven Effekt auf den Wiedereingliederungserfolg zu untersuchen.

## Abstract (en)

**Objectives:**

Returning to work often fails due to conflicting expectations and goals of the stakeholders involved. Due to their medical expertise and workplace-related knowledge, occupational physicians could effectively promote mutual understanding and cooperation between the stakeholders. The study aimed to develop a manual and training session for its application that will support occupational physicians to assume a mediating role in the integration of employees with a mental illness.

**Methods:**

Based on a literature review and extensive qualitative preliminary work on the expectations of the stakeholders, the manual and training materials were developed, discussed and revised in several consecutive steps based on a participatory approach with occupational physicians and other experts. Finally, occupational physicians were trained to use the manual during their specialization training in order to subsequently test its practicability in everyday work.

**Results:**

The manual presents information on the potentially different expectations of the actors for the return process and offers comprehensive advice for the mediating work of the occupational physicians. After a manualized training of approximately 2h, 9 out of 37 participants used the manual for return interviews within 4 months and 6 out of 9 rated it as helpful.

**Conclusion:**

The first positive experiences of using the material developed with the target group justify a larger interventional study to investigate the presumed beneficial effects on the success of reintegration.

Psychische Störungen wie Depressionen und Angststörungen sind Risikofaktoren für langfristige Arbeitsunfähigkeit und einen vorzeitigen Renteneintritt [1, 6, 21]. Wie eine dauerhafte Rückkehr an den Arbeitsplatz gelingen kann, bleibt – trotz umfangreicher Forschung – ein ungelöstes Problem [10, 18]. In Deutschland wurde 2004 das betriebliche Eingliederungsmanagement (BEM) gesetzlich eingeführt, welches die Rückkehr von Arbeitnehmer*innen mit schweren bzw. langdauernden Erkrankungen, wie z. B. psychischen Störungen, erleichtern soll (vgl. § 167 Abs. 2 SGV IX; [14]). Dieses formale Verfahren wird aber nur knapp mehr als einem Drittel der Arbeitnehmer*innen mit gesetzlichem Anspruch tatsächlich angeboten [8].

Betriebsärztinnen und Betriebsärzte (BÄ) sind im Prinzip mit ihrer medizinischen Expertise, der ärztlichen Schweigepflicht und detaillierten Kenntnissen über die individuellen Arbeitsbedingungen prädisponiert, den Rückkehrprozess vermittelnd zu unterstützen [21]. Sie stehen als Ansprechpartner*innen auch außerhalb des formalen BEM-Prozesses zur Verfügung [2].

So konnte z. B. in den Niederlanden gezeigt werden, dass die Einbeziehung von BÄ in den Rückkehrprozess positiv mit der Reduktion depressiver Symptome der Rückkehrenden assoziiert war [19]. Eine Kombination von arbeitsbezogenen und klinischen Interventionen im Vergleich zu alleinigen klinischen Interventionen ist mit einer verringerten Anzahl von Krankheitstagen assoziiert. Dieses Vorgehen wird bislang unzureichend umgesetzt [10]. Die Verwendung von – die Umsetzung fördernden – Leitlinien zur Wiedereingliederung psychisch erkrankter Beschäftigter durch BÄ führte in internationalen Studien zu uneinheitlichen Ergebnissen, was weiteren Forschungsbedarf begründet [3, 16, 17]. Im deutschen Bereich nimmt ein Leitfaden für BÄ der Deutschen Gesetzlichen Unfallversicherung (DGUV) deren Rolle im BEM im Allgemeinen in den Blick, lässt aber die spezifischen Problematiken bei der Rückkehr mit/nach psychischer Erkrankung außer Betracht [4].

Die berufliche Wiedereingliederung erfordert eine enge Kooperation verschiedener medizinischer, psychotherapeutischer und betrieblicher Akteure und Akteurinnen [10]. Bereits zwischen der medizinischen Regelversorgung und BÄ mangelt es an einer effektiven Kooperation [15]. An der Schnittstelle zwischen medizinischer inkl. psychotherapeutischer Versorgung und Betrieb ist es besonders notwendig, dass sich die beteiligten BÄ mit der Sichtweise, den Erwartungen und den Zielen anderer behandelnder Personen [13, 22] und betrieblicher Akteure und Akteurinnen auseinandersetzen. In einer vorausgehenden multiperspektivischen qualitativen Studie haben wir Sichtweisen und Erwartungen von psychisch erkrankten Beschäftigten, BÄ, Psychotherapeuten bzw. Psychotherapeutinnen und betrieblichen Akteure exploriert und gegenübergestellt [12]. Von allen Akteuren wurde die Schlüsselrolle der BÄ im Wiedereingliederungsprozess hervorgehoben [12].

## Zielstellung

Das Ziel der vorliegenden Studie besteht in der Entwicklung eines Instruments, unter Partizipation wesentlicher Akteure, welches die Kompetenzen von BÄ bei der Wiedereingliederung psychisch erkrankter Beschäftigter stärkt und sie dazu befähigt, eine vermittelnde und koordinierende Rolle einzunehmen. Dieses Ziel soll durch (1) die Entwicklung eines Leitfadens sowie (2) die Erstellung eines Schulungsmoduls zu seiner Anwendung im Rahmen der betriebsärztlichen bzw. arbeitsmedizinischen Fort- und Weiterbildung erreicht werden. Schließlich soll (3) in einer Pilotstudie geprüft werden, wie das Instrument von der Zielgruppe angenommen wird.

Im Folgenden wird die Entwicklung des Leitfadens und des Schulungsmoduls chronologisch dargestellt. Der gesamte Entwicklungszeitraum umfasste zwei Jahre. Die Ethikkommission der Heinrich-Heine-Universität Düsseldorf hat der Durchführung der vorliegenden Studie ein positives Votum erteilt (Studienregistriernummer: 6210R).

## Methodik und Ergebnisse

### Schritt 1: Qualitative Studie

#### Methodik.

Zwischen August 2018 und März 2019 wurden mit 68 Personen aus 5 Akteursgruppen leitfadengestützte Interviews zu ihren Erwartungen an die jeweils anderen und zu möglichen Konfliktsituationen durchgeführt und inhaltsanalytisch ausgewertet: rückkehrende Beschäftigte nach mehr als 6 Wochen Arbeitsunfähigkeit aufgrund einer psychischen Erkrankung (*n* = 24), deren Psychotherapeuten/Psychotherapeutinnen und Psychiater*innen (*n* = 13), BÄ (*n* = 13) sowie Beschäftigte mit (*n* = 9) und ohne (*n* = 9) Führungsverantwortung. Eine umfassende Darstellung der Methoden und Ergebnisse ist einer anderen Publikation zu entnehmen [12].

#### Ergebnisse.

Aus divergierenden Erwartungen wurden folgende potenzielle Konfliktsituationen identifiziert: Offenlegung der Erkrankung, Mitteilung über voraussichtliche Erkrankungsdauer und Arbeitsunfähigkeitszeit, Schonfrist mit reduzierter Arbeitslast, gestufte Wiedereingliederung, Nachbetreuung durch Vorgesetzte, Wiederaufnahme ins Team und gewünschter Arbeitsplatzwechsel. Allseitig wurde die Notwendigkeit wahrgenommen, die Kommunikation und Kooperation der beteiligten Akteure zu verbessern. Aufgrund der arbeitsplatzbezogenen Kenntnisse, der medizinischen Expertise und Schweigepflicht wurde die Übernahme einer Vermittlerrolle durch BÄ empfohlen [12].

### Schritt 2: Kognitive Interviews

#### Methodik.

Im Anschluss (September 2019) wurden telefonisch oder im persönlichen Kontakt kognitive Interviews mit einzelnen Interviewteilnehmer*innen, einem rückkehrenden Beschäftigten, 3 Psychotherapeuten bzw. Psychotherapeutinnen sowie 3 Beschäftigten mit und ohne Führungsverantwortung durchgeführt, um zu überprüfen, ob die vom Studienteam nach qualitativer Inhaltsanalyse zusammengefassten Erwartungen der jeweiligen Akteure aus deren Sicht das wiedergeben, was die Befragten tatsächlich ausdrücken wollten.

#### Ergebnisse.

Die Überprüfung der abgeleiteten Ergebnisse aus der qualitativen Studie im Rahmen der kognitiven Interviews ergab keine Änderungswünsche der Teilnehmer*innen. Die Ergebnisse und deren Darstellung spiegeln demnach mit hoher Wahrscheinlichkeit die zentralen Sichtweisen der Befragten wider.

### Schritt 3: Entwicklung von Lösungsvorschlägen

#### Methodik.

Da die Ergebnisse der qualitativen Studie potenziell divergierende Erwartungen zwischen den Akteuren im Rückkehrprozess zeigten und diese zu Konflikten bei der Wiedereingliederung führen können, wurden im September 2019 BÄ in einer Online-Studie bundesweit über die Mailing-Liste ArbMedNet^1^ angeschrieben und mit Hilfe von Freitextfeldern nach Lösungsvorschlägen zu sieben aus den Interviews hervorgegangenen Problemszenarien befragt. Beispielhaft kann hier das folgende Szenario genannt werden: In unserer Studie gaben die Beschäftigten oftmals an, dass sie ihre psychische Erkrankung am Arbeitsplatz nicht offenlegen wollen, da sie Stigmatisierung befürchten. Vorgesetzte und Kollegen wünschten sich hingegen oftmals, über die konkrete Erkrankung informiert zu werden, da sie der Ansicht waren, durch diese Kenntnis mehr Verständnis aufbringen zu können. Zudem gaben Vorgesetzte an, dass eine gezieltere Unterstützung bzw. Anpassung des Aufgabenspektrums bei Kenntnis der Erkrankung möglich sei. Die befragten BÄ konnten zu insgesamt 7 Problemszenarien ihre Vorgehensweise bzw. Lösungsvorschläge in Freitextfeldern angeben. Für die Studienteilnahme wurde eine Vergütung von 50 € angeboten.

#### Ergebnisse.

An der Studie nahmen 63 BÄ teil. Die Auswertung der Antworten erfolgte als Zusammenfassungen in Form von Stichpunkten, die als ausformulierte Lösungsvorschläge in den Leitfaden einflossen (Schritt 4). Diese Maßnahmen untergliedern sich in direkte Maßnahmen der BÄ, Hinweise zur Moderation zwischen Beschäftigten und Arbeitgebern, Maßnahmen zur Unterstützung des Arbeitsgebers, Maßnahmen im Betrieb sowie Maßnahmen durch weitere Beteiligte^2^.

### Schritt 4: Entwicklung des Leitfadens

#### Methodik.

**Figure 1:**
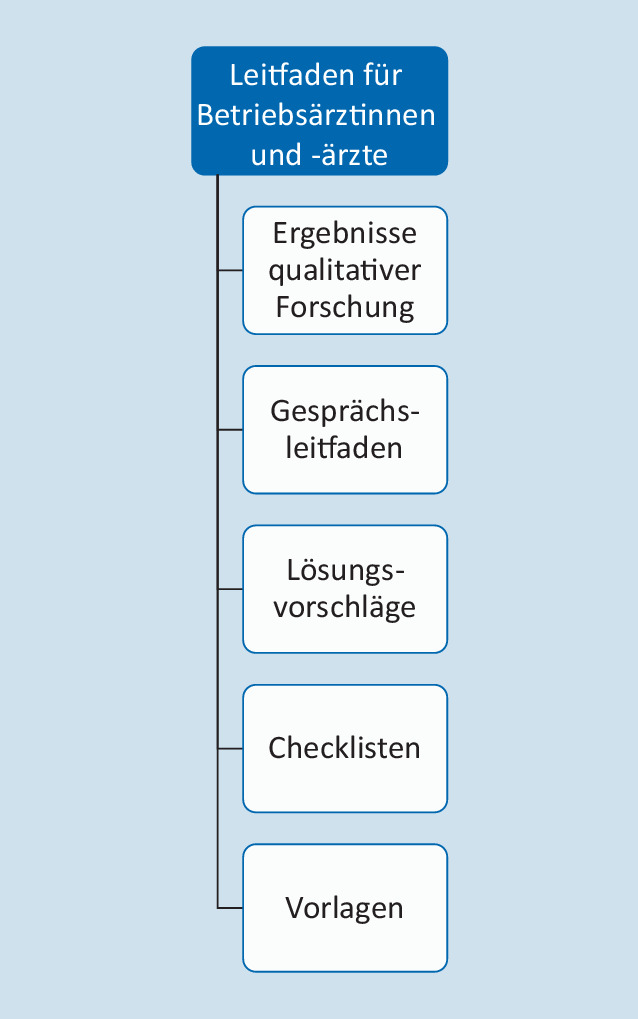

Ziel des Leitfadens ist die Unterstützung des betriebsärztlichen Einflusses auf einen gelingenden Rückkehrprozess nach längerer psychischer Erkrankung, d. h., spätestens nach 6‑wöchiger Arbeitsunfähigkeit innerhalb von 12 Monaten, wenn ein BEM-Verfahren eingeleitet werden müsste. Der Leitfaden kann allerdings auch schon früher eingesetzt werden und ist nicht an die Durchführung eines BEM-Verfahrens gekoppelt. Zentrales Element ist die Ermittlung von Wünschen und Erwartungen seitens des rückkehrenden Beschäftigten und der Vorgesetzten durch die BÄ. Basierend auf der internationalen wissenschaftlichen Literatur sowie den Ergebnissen vorheriger qualitativer Interviews [12] wurde ein Leitfaden entwickelt, der die in Abb. 1 dargestellten Elemente enthält. Neben den o. g. Ergebnissen der Schritte 1–3 wurden Checklisten und Vorlagen aus Materialien praktisch tätiger BÄ nach deren Empfehlung zusammengestellt. Zusätzlich wurde eine Anleitung für das Gespräch zwischen BÄ und rückkehrenden Beschäftigten entwickelt, da dies ein zentrales Element im Rückkehrprozess darstellt, sowie zwischen BÄ und weiteren Akteuren, z. B. Vorgesetzen. An der Entwicklung waren u. a. sehr erfahrene sowie auch noch in der Weiterbildung befindliche Arbeitsmediziner*innen mit und ohne praktische Erfahrung in Rückkehrprozessen beteiligt sowie Psycholog*innen und Gesundheitswissenschaftler*innen mit umfassender Erfahrung hinsichtlich der Übertragung qualitativer Befunde in standardisierte Instrumente [7, 20].

#### Ergebnisse.

Der Leitfaden zur Wiedereingliederung von Beschäftigten nach psychischer Erkrankung erläutert einführend Strukturen des Wiedereingliederungsprozesses und die Rolle des Betriebsarztes/der Betriebsärztin. Neben Hinweisen zum Datenschutz sowie zur Besonderheit bei psychisch erkrankten Beschäftigten wird in die Entstehung und den Gebrauch des Leitfadens eingeführt. Es folgen die Ergebnisse der umfangreichen qualitativen Vorarbeiten [12], zunächst in zwei Schaubildern, welche die anschließende textliche Darstellung zusammenfassen.

Das Kernelement des Leitfadens ist die auf Basis der qualitativen Vorarbeiten entwickelte dreiseitige Gesprächsanleitung, die darauf ausgerichtet ist, BÄ bei der Führung eines Erstgesprächs mit Beschäftigten zu unterstützen. Sie wird ergänzt durch ein Formular, welches von dem Beschäftigten bereits vor dem Gespräch ausgefüllt werden kann und ggf. den BÄ hilft, sich vorab zu informieren. Die Anleitung für das Erstgespräch zwischen BÄ und Rückkehrer wurde in Form einer Tabelle mit 3 Spalten gestaltet, die (1) relevante Themen aufführen (Tab. 1), (2) zu jedem Thema Formulierungsbeispiele für Fragen bereitstellen (Tab. 2) und (3) bei jeder Frage Raum für Notizen lassen^3^. Die Anleitung kann somit variabel genutzt werden, z. B. als ausformulierter Fragetext oder als Checkliste vor, während oder zum Abschluss des Gesprächs, sodass keine wichtigen Aspekte vergessen werden.

**Table 1:** 

Thema
Vorinformation der betroffenen Person zum Betrieblichen Eingliederungsmanagement (BEM)
Vorangegangene Gespräche mit anderen
Wünsche an dieses Gespräch
Schwerbehinderung
Derzeitige psychologische Betreuung
Aktueller Gesundheitszustand
Wunsch nach Frühberentung
Selbsteinschätzung: Rückkehr an den alten Arbeitsplatz möglich?
Ggf. Arbeitsplatzwechsel gewünscht?
Potenziell gesundheitsschädliche Arbeitsbedingungen am alten Arbeitsplatz
Rückkehr mit gestufter Wiedereingliederung
Umfang der Weiterbeschäftigung
Änderungswünsche bzgl. Arbeitsbedingungen
Offenlegung der Erkrankung
Wünsche der Vorgesetzten an Mitarbeiter bzw. vice versa
Kontaktaufnahme mit dem BEM-Team
Kontaktaufnahme mit Vorgesetzen
Kontaktaufnahme mit Psychotherapeuten/Psychiatern
Vereinbarung weiterer Gespräche

**Table 2:** 

Thema (Checkliste)	Fragen an die rückkehrende Person (Formulierungsbeispiele)
Vorinformationen der betroffenen Person zum Betrieblichen Eingliederungsmanagement (BEM)	Wieso sind Sie heute bei mir? Welche Informationen liegen Ihnen bereits zu diesem Gespräch oder zu Ihrer Rückkehr vor?
Vorangegangene Gespräche mit anderen Akteuren	Welche Gespräche bzgl. Ihrer Rückkehr an den Arbeitsplatz haben bereits stattgefunden?
Wünsche an dieses Gespräch	Was wünschen Sie sich von diesem Gespräch?
Schwerbehinderung	Wurde bei Ihnen eine Schwerbehinderung festgestellt?
Derzeitige psychologische Betreuung	Erhalten Sie (bereits/noch) Unterstützung von einem Psychotherapeuten/Psychiater?
Aktueller Gesundheitszustand	Mit Hinblick auf Ihre Erkrankung – wie werden Sie Ihre beruflichen Aufgaben bewältigen können?
Wunsch nach Frühberentung	Können Sie sich vorstellen, Ihren jetzigen Beruf/Ihre jetzige Tätigkeit für längere Zeit (Jahre), ggf. bis zu Ihrer Altersrente auszuüben?

Um Divergenzen zwischen maßgeblichen Akteuren möglichst frühzeitig aufdecken zu können, enthält der Leitfaden eine weitere – bzgl. Inhalt, Aufbau und Format analoge – Anleitung für das Gespräch zwischen dem Betriebsarzt/der Betriebsärztin und dem Vorgesetzten. Durch die identische inhaltliche Strukturierung und Formatierung der beiden Anleitungen können BÄ beide Sichtweisen leicht vergleichen, sofern mit beiden Parteien ein Gespräch geführt werden kann. Besprochene Maßnahmen können in einer beigefügten Übersichtstabelle aufgeführt und zusammen mit Verantwortlichkeiten und Fristen festgehalten werden.

Für den Fall divergierender Sichtweisen des Beschäftigten und dessen Vorgesetzten und somit potenzieller Konflikte sind die Lösungsvorschläge aus der o. g. Online-Studie (vgl. Schritt 3) aus der praktischen Arbeit erfahrener BÄ aufgeführt. Dieses Inventar von Lösungsvorschlägen kann erste Hinweise zur Konfliktlösung bieten, erhebt jedoch keinen Anspruch auf Vollständigkeit.

Des Weiteren beinhaltet der Leitfaden diverse Checklisten zur Orientierung in den betrieblichen Strukturen zur Wiedereingliederung, zur Kontaktaufnahme mit Behandelnden, ein Dokument zur Schweigepflichtentbindung, ein Muster für Anschreiben an Beschäftige zur Information über Rolle, Tätigkeit, Verantwortung und Möglichkeiten der BÄ im Rückkehrprozess, ferner Musteraushänge im Unternehmen, in denen BÄ auf ihre Tätigkeit hinweisen können. Der Leitfaden endet mit Literaturhinweisen.

### Schritt 5: Vorstellung des Leitfadens im Rahmen von Weiterbildungskursen für Arbeitsmediziner*innen und Betriebsärztinnen und -ärzte

#### Methodik.

Für die Einführung in den Leitfaden, seine praktische Nutzung und die Anwendung der Gesprächsanleitung wurde vom Studienteam eine Unterrichtseinheit entwickelt. Diese wurde im November 2019 in zwei Weiterbildungskursen pilotiert, um die praktische Durchführbarkeit der Unterrichtseinheit sowie die Akzeptanz seitens der Zielgruppe zu erfassen und Änderungsvorschläge einzuholen. Dies geschah einerseits im Rahmen des in der Weiterbildungsordnung vorgeschriebenen Kurses zur Erlangung der Facharztbezeichnung Arbeitsmedizin bzw. Zusatzbezeichnung Betriebsmedizin in Düsseldorf mit 25 Teilnehmenden sowie in einem Weiterbildungskurs für Arbeitsmedizinerinnen bzw. Arbeitsmedizinern und BÄ zur fachgebundenen Psychotherapie mit 20 Teilnehmenden. In der Unterrichtseinheit wurde der Leitfaden vorgestellt und die Gesprächsanleitung in einer Gesprächssimulation in Kleingruppen erprobt. Um zu ermöglichen, dass auch andere Expertinnen und Experten die Unterrichtseinheiten standardisiert verwenden können, wurde ein Schulungsmanual entwickelt, das frei verfügbar ist.^4^ Auch auf die dazugehörigen Präsentationsfolien kann zugegriffen werden.^5^

#### Ergebnisse.

Die Schulung besteht aus 2 Unterrichtseinheiten à 45 min. Sie beginnt mit der gesetzlichen Grundlage zum BEM sowie zur Studienlage zur Wiedereingliederung psychisch erkrankter Beschäftigter. Darauf folgen die Darstellung der Ergebnisse zu den Erwartungen verschiedener am Wiedereingliederungsprozess beteiligter Akteurinnen und Akteure aus den qualitativen Vorarbeiten sowie ein kurzer Überblick über Inhalt, Struktur und Anwendung des Leitfadens. Im Anschluss wird die Gesprächsanleitung für das Erstgespräch zwischen Betriebsärztin bzw. -arzt und rückkehrendem Beschäftigten vorgestellt und in Gesprächssimulationen praktisch eingesetzt. Dafür haben sich Kleingruppengrößen von ca. 6 Personen als praktikabel erwiesen. Es wurden vier verschiedene Varianten der Gesprächssimulation entwickelt, die in Abhängigkeit der Kursgröße, der zeitlichen Ressourcen sowie räumlichen Gegebenheiten eingesetzt werden können (s. Link zum Schulungsmanual oben).

Anhand der Rückmeldungen von Gruppendiskussionen am Ende der Unterrichtseinheiten wurde auch die Gesprächsanleitung final überarbeitet. Änderungen, die den Diskussionsbeiträgen der Teilnehmer*innen folgten, betrafen sensible Themen (bspw. mögliche angestrebte Frühberentung), die behutsam und ggf. zu einem späteren Zeitpunkt im Gesprächsleitfaden aufgegriffen werden sollten, wenn bereits eine Vertrauensbasis geschaffen werden konnte.

### Schritt 6: Praxiserprobung des Leitfadens

#### Methodik.

Nach Durchführung der beiden Schulungen und der Optimierung des Leitfadens wurde die Tauglichkeit der Unterrichtseinheit inklusive des ausgehändigten Leitfadens und der angeleiteten Gesprächssimulation im Praxiseinsatz überprüft. Dazu wurde die Schulung im Januar und Februar 2020 in 4 Weiterbildungskursen für Arbeitsmedizinerinnen und Arbeitsmedizinern und BÄ in Düsseldorf, Hamburg, Ulm und München mit insgesamt 95 Teilnehmenden durchgeführt. Anschließend wurden die Teilnehmenden gebeten, den Leitfaden und die Gesprächsanleitung in den kommenden Wochen, sofern indiziert und möglich, einzusetzen und einer Nachbefragung zuzustimmen. Insgesamt gaben 88 (93 %) Personen dazu ihr Einverständnis. Etwa 4 Monate nach Erhalt des Leitfadens konnten sich die Teilnehmer im Zeitraum vom 22. Juni bis zum 5. Juli 2020 an der Online-Nachbefragung zum Einsatz des Leitfadens bzw. der Gesprächsanleitung beteiligen. Für die Studienteilnahme wurde eine Aufwandsentschädigung in Höhe von 50 € angeboten.

#### Ergebnisse.

An der Pilotstudie zur Überprüfung der Praktikabilität beim Einsatz des Leitfadens und der Gesprächsanleitung nahmen insgesamt 37 von 88 Teilnehmenden aus allen 4 Weiterbildungsorten teil (Teilnahmequote: 42 %). Lediglich 9 Teilnehmer haben während des 4‑monatigen Nachbeobachtungszeitraums ein oder mehrere Wiedereingliederungsgespräche mit einem psychisch erkrankten Beschäftigten geführt und somit die Möglichkeit gehabt, den Leitfaden einzusetzen und seinen Nutzen zu bewerten. Von diesen 9 Betriebsärzten und Betriebsärztinnen gaben 7 an, den Leitfaden bzw. die Gesprächsanleitung eingesetzt zu haben. Insgesamt bewerteten 6 Befragte das Instrument als (sehr) hilfreich. Weitere 6 Personen waren (sehr) zufrieden. Fünf der Befragten gaben an, dass der rückkehrende Beschäftigte vom Einsatz des Leitfadens und der Gesprächsanleitung durch den Betriebsarzt bzw. die Betriebsärztin profitiert hat. Insgesamt 7 BÄ würden den Leitfaden weiterempfehlen.

Mittels Freitextfeldern wurden weitere Überarbeitungshinweise sowie Gründe für und gegen den Einsatz des Leitfadens eruiert. Aufgrund der Anmerkungen waren keine weiteren grundlegenden Überarbeitungen notwendig. Als Hinderungsgründe für den Einsatz des Leitfadens wurde angegeben, dass dieser vergessen oder aufgrund der Ausführlichkeit nicht eingesetzt wurde. Als Gründe für den Einsatz wurden die Vorbereitung sowie die Strukturierung des Gesprächs genannt und das Ziel, keine wichtigen Aspekte zu vergessen. Die befragten BÄ empfanden die Gesprächsanleitung als hilfreich, da sie selbst noch unerfahren waren, die angeleitete Vorbereitung zu einem sicheren Auftreten verholfen hat und ein Hineinversetzen in die Wünsche des Arbeitnehmers sowie des Arbeitgebers durch die Schaubilder zu Beginn des Leitfadens ermöglicht wurde. Ein Betriebsarzt beschreibt den Nutzen des Leitfadens folgendermaßen: „Zunächst scheint es mit mehr Zeitaufwand verbunden, aber im Nachhinein ist Gegenteiliges der Fall. Mit der Zeit macht sich der Leitfaden vielleicht auch überflüssig, sicher lohnt es sich aber auch dann noch, immer mal wieder nachzulesen. Ich bin froh, auf diese Hilfestellung zurückgreifen zu können.“

## Diskussion

Nach unserem Kenntnisstand ist der vorliegende Leitfaden für die Rückkehr von Beschäftigten mit psychischer Erkrankung an den Arbeitsplatz der erste, der multiperspektivisch (d. h. unter Einbeziehung von Beschäftigten mit psychischen Erkrankungen, Psychotherapeuten/Psychiatern sowie dem sozialen Umfeld am Arbeitsplatz) und partizipativ mit Betriebsärzten und Betriebsärztinnen für Betriebsärzte und Betriebsärztinnen entwickelt worden ist und der speziell auf die Erhebung potenziell divergierender Erwartungen verschiedener am Rückkehrprozess beteiligter Akteure abzielt. Zudem bietet der Leitfaden Lösungsvorschläge zu möglichen Konflikten sowie weitere unterstützende Materialien für BÄ. Dadurch kann die vermittelnde Position der BÄ im Rückkehrprozess gestärkt werden. Zudem basiert der Leitfaden auf aktuellen wissenschaftlichen Erkenntnissen zur Wiedereingliederung psychisch erkrankter Beschäftigter sowie auf umfangreichen qualitativen Vorarbeiten mit verschiedenen Akteuren des Wiedereingliederungsprozesses. Ein zusätzlicher Vorzug ist die Schulung, die die Verbreitung der Materialien im Rahmen der Weiterbildung fördert und den praktischen Einsatz des Leitfadens vermittelt. Sowohl der Leitfaden als auch die Schulung (Manual und Folien) sind frei verfügbar (Links – s. oben).

Ausgehend von der ungenügenden Umsetzung des BEM [8] und der wahrgenommenen Unsicherheit bezogen auf den Umgang mit psychischen Erkrankungen in Unternehmen [5] sind strukturierte Wiedereingliederungsprozesse notwendig, um die Implementierung voranzutreiben. Da die betriebsärztliche Betreuung von Unternehmen gesetzlich vorgeschrieben ist und BÄ sowohl medizinische als auch arbeitsplatzbezogene Kenntnisse aufweisen, ist die Verortung der Wiedereingliederung als betriebsärztliche Aufgabe sinnvoll [19]. Der entwickelte Leitfaden soll BÄ unterstützen, insbesondere in der Initiierung und zu Beginn eines Rückkehrprozesses diese Tätigkeit in Eigeninitiative zu übernehmen. In einer Studie in den Niederlanden wurde festgestellt, dass die Befolgung einer nationalen Leitlinie zur Eingliederung von psychisch erkrankten Beschäftigten durch BÄ mit einer frühzeitigeren Rückkehr des Beschäftigten assoziiert ist und damit die strukturierte Durchführung von Eingliederungsprozessen zu empfehlen ist [11]. In einer weiteren niederländischen Studie wurde die Notwendigkeit beschrieben, dass BÄ in Bezug auf den Einsatz von Leitfäden geschult werden sollten, um die konkrete Anwendung in der Praxis zu fördern [9]. Die im Rahmen dieser Studie entwickelten Leitfäden und Schulungsmaterialien setzen an dieser Forderung an. Grundsätzlich sind der tatsächliche Einsatz des Leitfadens sowie die Einbindung der BÄ in einen BEM-Prozess jedoch weitgehend von der Eigeninitiative des einzelnen Arztes und dem jeweiligen betrieblichen Gesundheitsmanagement abhängig, da die Einbindung in den BEM-Prozess in Deutschland optional ist und auf Wunsch des rückkehrenden Beschäftigten erfolgt. Zudem ist die Wahrscheinlichkeit, ein formales BEM-Verfahren angeboten zu bekommen, stark abhängig von der Größe des Unternehmens, in dem die Betroffenen beschäftigt sind: Je größer das Unternehmen, desto eher ist ein BEM implementiert und wird den Beschäftigten angeboten [8]. Die Förderung der Umsetzung des BEM in Klein- und Kleinstunternehmen ist daher erforderlich. Aufgrund dieser bislang unzureichenden generellen Umsetzung des BEM liegt eine besondere Stärke des Leitfadens darin, dass er explizit auch außerhalb eines offiziellen Verfahrens eingesetzt werden kann. Des Weiteren ist die Einbeziehung eines breiten Spektrums von BÄ bei der partizipativen Entwicklung und Optimierung des Leitfadens hervorzuheben. Es wurden BÄ involviert, die sich noch in der arbeitsmedizinischen Weiterbildung befinden, sowie bereits sehr erfahrene, die sich speziell in der psychotherapeutischen Versorgung von Beschäftigten weiterbilden. Durch die Kopplung des Leitfadens mit einem didaktischen Konzept (Schulungsmodul/Unterrichtseinheit und Schulungsmanual) werden vor allem die Verbreitung und der konkrete Einsatz des Instruments gefördert. Zudem unterstützt der Leitfaden nicht nur die Gesprächsführung und Aufdeckung von potenziellen Konfliktpunkten im Rahmen der Wiedereingliederung, sondern bietet erste Lösungsvorschläge, welche von erfahrenen BÄ erstellt wurden. Diverse Schaubilder und Checklisten stellen ein zusätzliches, hilfreiches Angebot dar.

Zu erwähnen sind dennoch methodische Limitierungen unserer mehrstufigen Entwicklungsarbeit. So war insbesondere die Praxiserprobung des Leitfadens (vgl. Schritt 7) erschwert, da teilnehmende BÄ nur nach vorheriger Schulung rekrutiert wurden und die Stichprobe somit klein war (*n* = 88). An der Nachbefragung zum Einsatz des Leitfadens nahmen nur 42 % teil, und nur wenige BÄ hatten überhaupt die Möglichkeit, ein Wiedereingliederungsgespräch nach psychischer Krankheit zu führen. Ein Selektionsbias ist aufgrund dieser Rücklaufquote nicht auszuschließen (z. B., wenn Nichtteilnehmende den Leitfaden kritischer bewerten würden). Ein Faktor, der die Teilnahme jedoch möglicherweise allgemein erschwert hat, ist die SARS-CoV-2-Pandemie, die erst nach Abschluss der Schulungen (Februar 2020) Auswirkungen auf Unternehmen hatte (Schließung, Kurzarbeit, Kontaktbeschränkungen) sowie möglicherweise die Rückkehr psychisch erkrankter Beschäftigter an den Arbeitsplatz erschwert hat (Kurzarbeit, Homeoffice etc.). In diesem Kontext könnte eine größer angelegte Studie über einen längeren Zeitraum zu aussagekräftigeren Ergebnissen führen. Zudem könnten in Zukunft auch Informationen über den Nutzen aus Sicht der rückkehrenden Beschäftigten erhoben werden, bei denen der Gesprächsleitfaden im Wiedereingliederungsprozess eingesetzt wurde. Darüber hinaus könnten auch Vorgesetzte befragt werden, und man könnte im Sinne einer Wirksamkeitsstudie prüfen, ob das BEM durch den Einsatz des Leitfadens tatsächlich erfolgreicher gestaltet werden kann (bspw. kürzere Dauer bis zur Rückkehr, geringere Rückfallquote). Zudem könnten auch behandelnde Therapeutinnen und Therapeuten sowie Ärztinnen und Ärzte des Rückkehrenden befragt werden, falls diese im Rahmen des BEM einbezogen werden. Da sich die der Leitfadenentwicklung zugrunde liegenden Daten vorwiegend auf Depressionen und Angststörungen beziehen, ist die Übertragbarkeit des Leitfadens auf andere psychisch bedingte Krankheitsgruppen nicht ohne weitere Untersuchungen möglich. Des Weiteren kann der Nutzen des Instruments für verschiedene primär somatische Erkrankungen überprüft werden. Insbesondere bei physischen Erkrankungen gibt es ggf. Themen im Leitfaden, die nicht oder weniger relevant sind (z. B. Kontakt zu Psychotherapeuten oder Offenlegung der Erkrankung) oder die noch fehlen. Dies könnte in einer entsprechenden Studie ermittelt werden. In Bezug auf die Unternehmensgröße, Berufsgruppen oder spezifische Settings wurde der Leitfaden so konzipiert, dass er auf verschiedenste berufliche Situationen übertragbar ist: Die zu verwendenden Kernfragen sind für den Rückkehrprozess relevant und weitgehend unabhängig vom betrieblichen Kontext.

## Fazit

Zusammenfassend konnte ein Leitfaden entwickelt werden, welcher auf den Erwartungen und Bedürfnissen relevanter am Eingliederungsprozess beteiligter Akteure basiert und diese abbilden kann. Durch das partizipative Vorgehen entstand ein Instrument, welches von der Zielgruppe akzeptiert sowie als hilfreich bewertet wird und somit mit größerer Wahrscheinlichkeit in der Praxis eingesetzt wird. Durch die Entwicklung eines Schulungsmoduls mit praktischer Übung zur Anwendung des Leitfadens wird der wissenschaftlichen Forderung nach einem entsprechenden Training zu entwickelten Leitfäden begegnet. Da ein Schulungsmanual entwickelt wurde, kann die Schulung unabhängig vom Studienteam, d. h. durch andere Kursleiter*innen mit arbeitsmedizinischem Hintergrundwissen und didaktischen Kompetenzen vorbereitet und durchgeführt werden. Somit sind der Einsatz der Schulung und die breite Dissemination der Leitfäden beispielsweise durch Lehrende in Weiterbildungskursen zum Facharzt für Arbeitsmedizin/Zusatzbezeichnung Betriebsmedizin möglich. Der Effekt dieses Maßnahmenbündels auf den Erfolg der Rückkehr an den Arbeitsplatz von Beschäftigten mit psychischen Erkrankungen sollte in einer randomisierten Interventionsstudie unter realen Bedingungen überprüft werden.

## Footnotes

1 Das ArbMedNet ist ein E‑Mail-Verteiler für den Informations- und Meinungsaustausch im Bereich der Arbeits- und Umweltmedizin sowie verwandter Gebiete, für den (Stand August 2020) 4013 Abonnenten mit vorwiegend arbeitsmedizinischem Berufshintergrund registriert waren: http://www.klinikum.uni-muenchen.de/Institut-und-Poliklinik-fuer-Arbeits-Sozial-und-Umweltmedizin/de/arbmednet/index.html.

2 Im Leitfaden zu finden auf den S. 32–39: https://www.uniklinik-duesseldorf.de/fileadmin/Fuer-Patienten-und-Besucher/Kliniken-Zentren-Institute/Institute/Institut_fuer_Arbeits_Sozial_und_Umweltmedizin/Materialien/Downloads/Handlungsleitfaden_RTW_nach_psychischen_Krisen_final.pdf.

3 Der Leitfaden ist online verfügbar unter: https://www.uniklinik-duesseldorf.de/fileadmin/Fuer-Patienten-und-Besucher/Kliniken-Zentren-Institute/Institute/Institut_fuer_Arbeits_Sozial_und_Umweltmedizin/Materialien/Downloads/Handlungsleitfaden_RTW_nach_psychischen_Krisen_final.pdf.

4 https://www.uniklinik-duesseldorf.de/fileadmin/Fuer-Patienten-und-Besucher/Kliniken-Zentren-Institute/Institute/Institut_fuer_Arbeits_Sozial_und_Umweltmedizin/Materialien/Downloads/Schulungsmanual_RTW_final.pdf.

5 https://www.uniklinik-duesseldorf.de/patienten-besucher/klinikeninstitutezentren/institut-fuer-arbeits-sozial-und-umweltmedizin/downloads.
